# MicroRNA MiR-490-5p suppresses pancreatic cancer through regulating epithelial-mesenchymal transition via targeting MAGI2 antisense RNA 3

**DOI:** 10.1080/21655979.2021.2024653

**Published:** 2022-01-19

**Authors:** Zhenglei Xu, Zeming Chen, Minsi Peng, Zhuliang Zhang, Weixiang Luo, Ruiyue Shi, Lisheng Wang, Yingcai Hong

**Affiliations:** a Department of Gastroenterology, Shenzhen People’s Hospital (The Second Clinical Medical College, Jinan University, the First Affiliated Hospital, Southern University of Science and Technology), Guangdong, China; b The Second Clinical Medical College, Jinan University, Guangdong, China; c Department of Nursing, Shenzhen People’s Hospital (The Second Clinical Medical College, Jinan University, the First Affiliated Hospital, Southern University of Science and Technology), Guangdong, China; d Department of Thoracic Surgery, Shenzhen People’s Hospital (The Second Clinical Medical College, Jinan University, the First Affiliated Hospital, Southern University of Science and Technology), Guangdong, China

**Keywords:** MicroRNA-490-5p, MAGI2-AS3, Epithelial–mesenchymal transition

## Abstract

Pancreatic cancer with about 5% five-year overall survival rate remains a challenge. Invasion and migration of pancreatic cancer cells are the main factors leading to poor prognosis. MicroRNA-490-5p (miR-490-5p) has anti-cancer effects in a variety of tumors, but its role in pancreatic cancer has not been reported. The mRNA expressions of miR-490-5p, MAGI2 antisense RNA 3 (MAGI2-AS3), Matrix metalloproteinase (MMP)2, MMP9, N-cadherin, and E-cadherin were detected by quantitative real-time PCR, while the protein expressions of these genes except miR-490-5p were measured by Western blot analysis. The cell viability, apoptosis, migration and invasion were detected by cell counting kit-8 (CCK-8), apoptosis and transwell assays. MiR-490-5p was abnormally low-expressed in pancreatic cancer, whose down-regulation generated enhanced effects on viability, migration and invasion in pancreatic cancer cells, as well as MAGI2-AS3 expression. MiR-490-5p mimic exerted the opposite effect on cells, which also down-regulated MMP2, MMP9, and N-cadherin protein expressions, while up-regulating E-cadherin protein expression. MAGI2-AS3, which was the targeted binding site of miR-490-5p, promoted viability, migration and invasion, and inhibited apoptosis of cancer cells. More importantly, miR-490-5p played an anti-cancer role in pancreatic cancer by targeting MAGI2-AS3 and regulating epithelial–mesenchymal transition (EMT), which was partially offset by MAGI2-AS3.

## Introduction

Pancreatic cancer, which ranks as the fourth most prevalent cause of cancer-related mortality in the western world with a 5-year survival rate of less than 5%, bears a high probability to be the second leading cause within a decade [1]. The main clinical manifestations of the disease refer to abdominal pain or discomfort, jaundice, weight loss, and persistent/intermittent fever [2,3]. Surgical resection is recognized as the only possible cure for pancreatic cancer, however, there are few patients (15 to 20%) qualified for excision due to locally progression or metastasis of most tumors at diagnosis, and five-year survival rate was about 20% in patients treated with successful resection and adjuvant, with 25 to 30 months median survival [4]. Gemcitabine is the preferred chemotherapy drug approved for the treatment of pancreatic cancer, but it is prone to drug resistance, leading to chemotherapy failure [2]. Therefore, new initiatives should be put forward and developed from multifaceted perspectives to care for pancreatic cancer.

In recent years, numerous studies have pointed to microRNAs (miRNAs) in that they were found to be involved in promoting or inhibiting the occurrence and development of various diseases. Previous studies revealed that miR-145, which was related to invasion in colorectal cancer, was lowly expressed in colorectal tumor [5]. A clinical study of 32 cases of healthy women, 163 cases of patients with epithelial ovarian cancer, and 20 cases of patients with ovarian benign on quantitative level of serum outside the body of miRNAs was conducted, the results indicated that miR-200b and miR-200 c exosomal levels were up-regulated which were related to epithelial ovarian cancer lymph node metastasis and International Federation of Gynecology and Obstetrics (FIGO) III-IV stage [6]. Another research proved that downregulation of miR-139-5p and miR-139-3p expression levels were closely related to the promotion of migration and invasion of bladder cancer cells [7].

MiRNA expression profile analysis revealed abnormal miRNA expression in serum and tumor tissues of pancreatic cancer patients was closely attributed to drug resistance, disease stage, or even survival of patients, so targeting these tiny molecules, known as specific miRNAs, could provide an effective and optimal method for the possible treatment for this disease [8]. Besides, nanoparticles delivery of synthetic oligonucleotides or treatment with natural medicines can regulate the expression of miRNAs, thus inhibiting the growth of pancreatic cancer, which prompts that targeted miRNAs in combination with traditional cancer treatments may be a new therapeutic strategy to improve drug sensitivity and achieve better curative effects for patients with pancreatic cancer [8]. We searched for reports pertaining to the correlation between miRNAs and digestive cancers and verified that 16 miRNAs, including miR-490 which locates on chromosome 7q33, were down-regulated in digestive cancers, pancreatic cancer, for instance [9]. Others have confirmed that microRNA-490-5p (miR-490-5p) expression was down-regulated in hepatocellular carcinoma tissues, the alteration of which in cancer cells by transfection could affect cell proliferation, migration and other biological behaviors [10]. However, to the best of our knowledge, there is a dearth of accessible literature regarding the correlation between miR-490-5p and pancreatic cancer.

The function of miR-490-5p and its underlying mechanism on pancreatic cancer are unknown, which therefore emerges as the purpose of this research, so as to provide a theoretical basis for the study and development of a novel therapeutic approach for the disease.

## Materials and Methods

### Patient samples

A total of 20 pancreatic cancer tissues and adjacent tissues were collected from 20 patients aging from 32 to 55, who diagnosed in the Second Clinical Medical College of Jinan University, Shenzhen People’s Hospital from May 2018 to April 2019. Inclusion criteria included resectable pancreatic cancer confirmed by imaging and biopsy, the absence of prior chemotherapy or radiotherapy, as well as normal renal function [11,12]. Exclusion criteria contained patients with metastatic pancreatic cancer, surgery, chemotherapy or other treatment, as well as in pregnancy/lactation [13]. Approval was obtained from the Second Clinical Medical College of Jinan University, Ethics Committee of Shenzhen People’s Hospital. Written informed consent was acquired from each patient. Ethics committee approval number is CH20185658.

### Cell culture

Human normal pancreatic duct epithelial cell HPDE6-C7, and pancreatic cancer cell lines PANC-1, SW1990, AsPC-1 and BxPC-3 were purchased from Shanghai Cell Bank (Shanghai, China). DMEM medium (11,965,084, Gibco, Waltham, MA, USA) containing 10% fetal bovine serum (FBS, 10,099,141, Gibco) and 1% penicillin-streptomycin (15,070,063, Gibco) was used to culture cells in a humidified atmosphere at 37°C with 5% CO_2_ [14].

### Grouping

In order to explore the function of miR-490-5p on cells, the cells were divided into groups as following: control group (untreated cells), inhibitor or mimic control group (cells transfected with miR-490-5p inhibitor control or mimic control), and inhibitor or mimic group (cells transfected with miR-490-5p inhibitor or mimic). For the rescue experiment, the cells were divided into groups as following: control group, negative control (NC) group (cells transfected with NC), MAGI2 antisense RNA 3 (MAGI2-AS3) group (cells transfected with pcDNA3.1-MAGI2-AS3 recombinant plasmids), MAGI2-AS3+ mimic group (cells transfected with miR-490-5p mimic and pcDNA3.1-MAGI2-AS3 recombinant plasmids), and mimic group.

### Transfection and dual-luciferase reporter assay

For transfection, the cells were transfected with miR-490-5p inhibitor (5ʹ-ACCCGCCUGGGGAGUAUCCAUGG-3ʹ), miR-490-5p mimic (5ʹ-CCAUGGUACUCCCCAGGCGGGU-3ʹ) as well as their corresponding NC plasmids, which were synthesized by GenePharma (Shanghai, China). The transfection was performed using Lipofectamine 2000 (11,668,019, Invitrogen, Carlsbad, CA). For plasmid construction, the HA MAGI2-AS3 E545K fragment derived from pBABE puro HA MAGI2-AS3 E545K was cloned into pcDNA3.1/V5-His-TOPO (K480001, Invitrogen, Carlsbad, CA), with the usage of Qiagen Plasmid Midi kit (12,191, Hilden, Germany) and QiaQuick Gel extraction kit (28,704). The pCMV2-Tag 2A MAGI2-AS3-WT was acquired from Addgene (MA, USA). The MAGI2-AS3 mutant 3ʹ-UTR was generated on the basis of MAGI2-AS3 3ʹ-UTR. The transfection was performed by FuGENE HD (#E2311, Promega Corporation, Madison, WI, USA). Further experiments were conducted 48 hours (h) after transfection. For dual-luciferase reporter assay [15], miR-490-5p mimic or blank vectors and wild-type or mutant MAGI2-AS3 3ʹ-UTR plasmids were co-transfected into cells by X-tremeGENE (06366236001, Roche, Penzberg, Germany). Dual-luciferase reporter system (E1910, Promega) was used to detect luciferase activity.

### Quantitative real-time polymerase chain reaction (qRT-PCR) analysis

The tissues were first cut into small pieces and placed in an ordinary glass homogenizer, and then added Trizol reagent (9109, Takara, Japan) according to the manufacturer’s instructions. Total RNAs from cells were extracted by Trizol reagent. Next, the cDNAs were synthesized with a reverse transcription kit (DRR047S, Takara). Then the amplification of qRT-PCR reaction was conducted using SYBR Premix Ex Taq kit (RR420, Takara). The condition for qRT-PCR was: 95°C for 5 s, and 40 cycles at 95°C for 15 s and 60°C for 15 s. The result of miRNA was normalized to U6 and GAPDH. The sequences of primers were as followed: miR-490-5p, forward: 5ʹ-CCATGGATCTCCAGGTGGGT-3ʹ; U6, forward 5ʹ-TGCGGGTGCTCGCTTCGGCAGC-3ʹ; MAGI2-AS3, F-5ʹ-GGCTCCGATGGAGCAGAAAT-3ʹ and R-5ʹ-CTGTCCTCCCCTCTCTTGGA-3ʹ; E-cadherin, F-5ʹ-TGCCCAGAAAATGAAAAAGG-3ʹ, and R-5ʹ-GTGTATGTGGCAATGCGTTC-3ʹ; N-cadherin, F-5ʹ-CCATCACTCGGCTTAATGGT-3ʹ, and R-5ʹ-ACCCACAATCCTGTCCACAT-3ʹ; GAPDH, F-5ʹ-TGCCAAATATGACATCAAGAA-3ʹ, and R-5ʹ-GGAGTGGGTG TCGTCGCTGTTG-3. PCR results were calculated by 2^−ΔΔCt^ method [16].

### Cell counting kit-8 (CCK-8) assay

After transfection for 48 h, cells (2 × 10^4^/well) were plated in every well of 96-well plates, and 10 μL CCK-8 solution (CK04, Dojindo, Tokyo, Japan) was added to each well at 24 h, 48 h and 72 h. Next, the cells were incubated for 1 h. The absorbance was detected at a wavelength of 450 nm using a microtiter plate (Immulon 4HBX, Thermo Labsystems, Rochester, NY) [17].

### Apoptosis assay

After transfection of 48 h, apoptosis of the cells was measured by a FACScan flow cytometer (BD Biosciences, USA). In this experiment, cells were stained using Annexin V-fluorescein isothiocyanate (Annexin V, 10 μL) (BMS500FI-300, Thermo Fisher Scientific Life Sciences, Waltham, MA) and propidium iodide (PI, 5 μL) as per the manufacturer’s protocol [18]. The percentage of Annexin V/PI cells was quantified using FACScan flow cytometer.

### Western blot analysis

The cell extracts were lysed with ice-cold lysis buffer. The protein concentration was calculated using BCA Protein Assay kit (23,227, Pierce) and equal amount of proteins were separated by SDS-PAGE, as described previously [17]. Then, the proteins were transferred onto a polyvinylidene fluoride (PVDF) membrane which was blocked for 1 h with 5% nonfat milk and incubated with primary antibodies against Matrix metalloproteinase (MMP)2 (1:1000, 74kD, ab215986, Abcam), MMP9 (1:1000, 78kD, ab219372, Abcam), E-cadherin (1:10,000, 97kD, ab40772, Abcam), N-cadherin (1:1000, 130kD, ab18203, Abcam) and GAPDH (1:10,000, 36kD, ab181602, Abcam) at 4°C overnight, followed by the incubation with secondary antibody goat anti-rabbit IgG H&L (1:1000, ab205718, Abcam) for 1 h. Then, the Image-Pro Plus 6.0 software (Media Cybernetics, Inc., MD, USA) was used to analyze proteins expressions. GAPDH was used as an internal control.

### Transwell assay

For migration detection, after transfection for 24 h, cells (3 × 10^5^/mL) were cultured in medium (no serum). The final concentration of fetal bovine serum was 1% and the volume was 150 μL when cells were added to the upper chamber. We added 10% FBS medium in the lower chamber to make the volume 500 μL. After 48 h of incubation in the cell incubator, the upper chamber fluid was absorbed and placed in a 24-well plate containing 500 μL of 4% paraformaldehyde (G1101, Servicebio, China). The cells were fixed at room temperature for 20 min. After imbibing the fixative solution, the cells were placed in a 24-well plate containing 500 μL 0.1% crystal violet solution (G1014, Servicebio) for 20 min, and then the inner surface cells of the bottom membrane of the upper ventricle were wiped off. The results were observed and captured under the microscope. For invasion measurement [19], the apical chamber was coated with Matrigel matrix and then cells were added. The remaining steps were the same as those in migration detection.

### Statistical analysis

Data were expressed as mean ± standard deviation and analyzed by GraphPad Prism 8 software (GraphPad Software Inc., San Diego, CA). Student *t*-test was used for analyzing the difference between the two groups, and one-way ANOVA was used for analyzing the difference among two more groups followed by Bonferroni test. *P* < 0.05 was indicative of a statistical significance.

## Results

In this article, the aim was to explore whether miR-490-5p could influence the hallmarks of pancreatic cancer including proliferation, migration, invasion, and epithelial–mesenchymal transition (EMT). We discovered that miR-490-5p mimic weakened the malignant phenotype of pancreatic cancer through regulating EMT via attenuating MAGI2-AS3, which offered a new entry point to the therapy of pancreatic cancer.

### MiR-490-5p in pancreatic cancer tissues and cells

**Figure 1.**
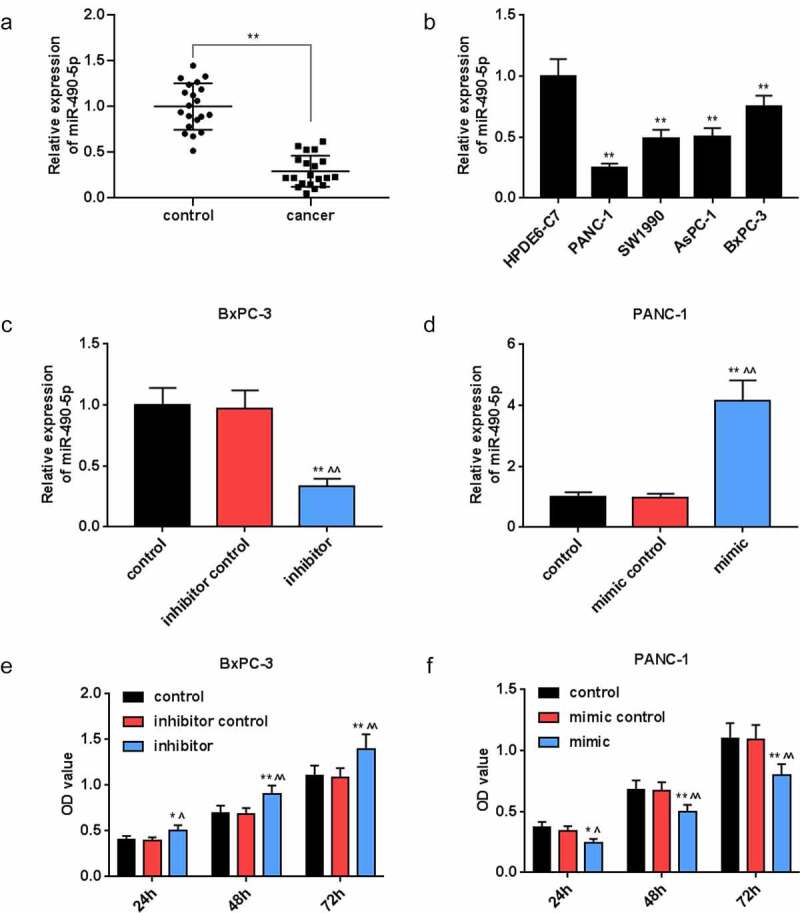
Expression of miR-490-5p in pancreatic cancer and its effect on the cell viability. (a) Expression of miR-490-5p in pancreatic cancer tissues was found to be lower than that in adjacent tissues, which was detected by quantitative real-time PCR (qRT-PCR). (b) The expression level of miR-490-5p in normal pancreatic duct epithelial cells HPDE6-C7 was significantly higher than that in pancreatic cancer cell lines PANC-1, SW1990, AsPC-1, and BxPC-3, which was detected by qRT-PCR. (c-d) The qRT-PCR was used to detect the level of miR-490-5p in cells to verify successful transfection. (e-f) Cell counting kit-8 (CCK-8) assay was used to detect cell viability. As the cell viability increased over time, the silencing of miR-490-5p inhibited cell viability, but the overexpression of miR-490-5p showed the opposite effect. *p < 0.05, **p < 0.001, vs. HPDE6-C7 or control; ^^^*p* < 0.05, ^^^^*p* < 0.001 vs. inhibitor control or mimic control.

For exploring the correlation between miR-490-5p and pancreatic cancer, we collected pancreatic cancer tissues and adjacent tissues from 20 clinical patients. The qRT-PCR analysis results documented that the expression of miR-490-5p in cancer group was remarkably lower than that in control group (Figure 1a, *p* < 0.01), while it was lowly expressed in pancreatic cancer cell lines PANC-1, SW1990, AsPC-1, and BxPC-3 in comparison with that in HPDE6-C7 cells (Figure 1b, *p* < 0.01).

### Detection of transfection efficiency

In view of the differential expression of miR-490-5p in collected pancreatic cancer patients and normal tissues, the effect of miR-490-5p expression on pancreatic cancer cells was further analyzed. Before that, the transfection rate was detected by qRT-PCR. The data showed that in BxPC-3 cells, the lowest expression of miR-490-5p was observed in inhibitor group compared with that in other groups (Figure 1c, *p* < 0.01), while in PANC-1 cells, the highest expression of miR-490-5p was observed in mimic group compared with that in other groups (Figure 1d, *p* < 0.01).

### Effect of miR-490-5p on cell biological behaviors

**Figure 2.**
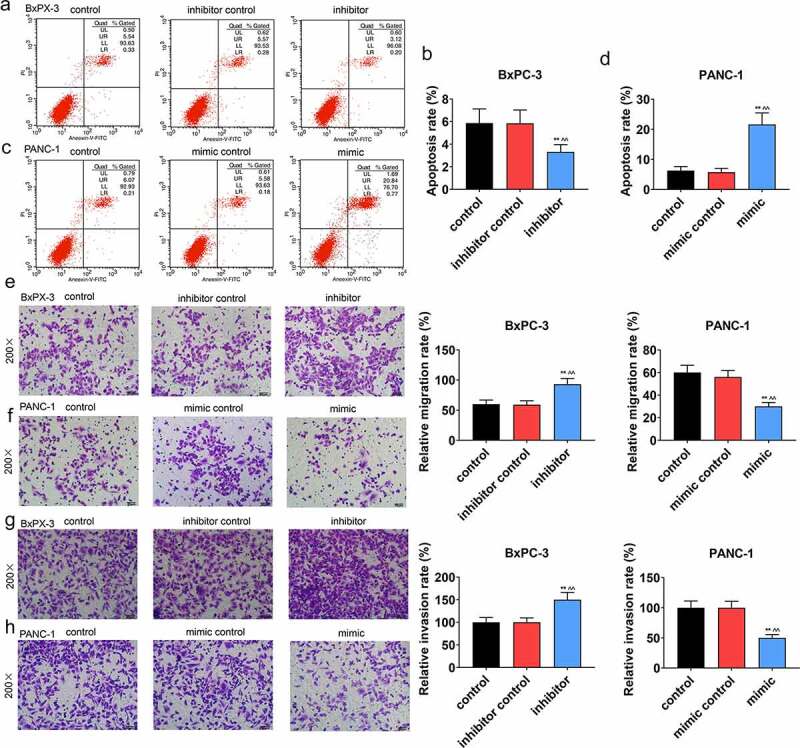
Effect of miR-490-5p on migration, invasion and apoptosis of cancer cells. (a-d) Detection of apoptotic rate by flow cytometry revealed that silencing of miR-490-5p reduced the apoptotic rates of cells, while overexpression of miR-490-5p showed the opposite effect. (e-f) Silencing of miR-490-5p in cells promoted migration, while overexpression of miR-490-5p did the opposite, which was detected by transwell experiment. (g-h) Silencing of miR-490-5p increased invasion, while overexpression of miR-490-5p did the opposite, which was detected by transwell experiment. **p < 0.001, vs. control; ^^*^*^*p* < 0.001 vs. inhibitor control or mimic control.

We conducted a series of experiments to explore the miR-490-5p function on cell biological behaviors. In CCK-8 assay, the data indicated that the optical density (OD) value at the wavelength of 450 nm in groups with pancreatic cancer cells, which received different treatments, was increased with time. Compared with other groups (Figure 1e, *p* < 0.05), in inhibitor group containing BxPC-3, the OD value was the highest; while in, mimic group containing PANC-1, the OD value was the lowest (figure 1f, *p* < 0.05). Based on apoptosis assay, in contrast with the apoptosis in other groups, a marked reduction in the apoptosis of the BxPC-3 cells was presented in inhibitor groups (Figure 2a-b, *p* < 0.01), while the opposite trend was observed in the apoptosis of the PANC-1 cells in mimic groups (Figure 2c-d, *p* < 0.01). Moreover, in transwell experiment, the data suggested that in BxPC-3 cells, the migration rate was predominantly promoted in inhibitor group in comparison with other groups (Figure 2e, *p* < 0.01), while in PANC-1 cells, the migration rate was inhibited in mimic group in comparison with other groups (figure 2f, *p* < 0.01). In addition, the results of invasion rate measuring signified that in BxPC-3 cells, among all groups, an increase of invasion rate was observed in inhibitor group (Figure 2g, *p* < 0.01), while in PANC-1 cells, the lowest invasion rate appeared in mimic group (Figure 2h, *p* < 0.01).

### The correlation between miR-490-5p and MAGI2-AS3

**Figure 3.**
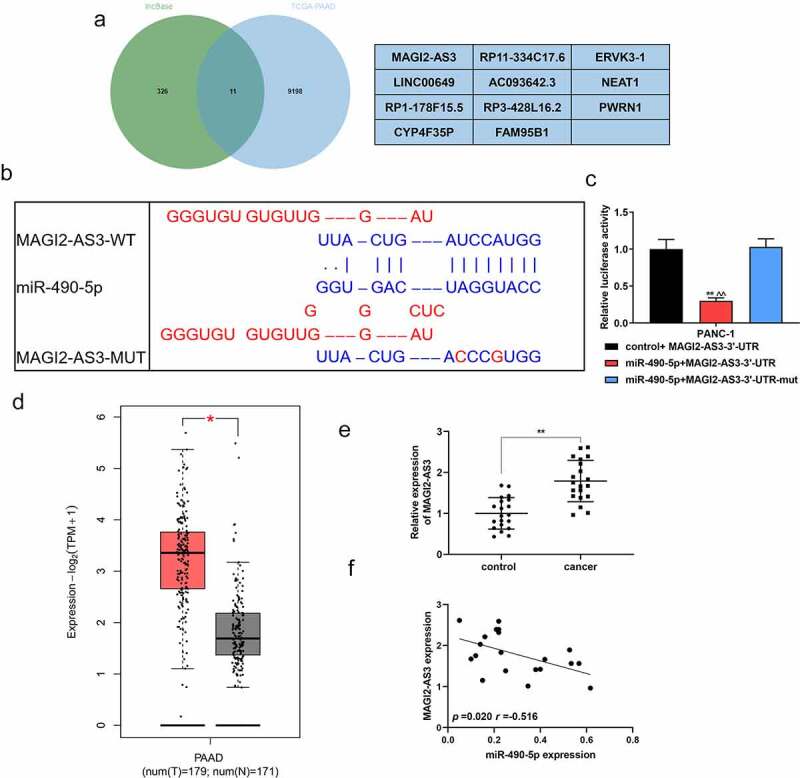
The targeting relation between MAGI2-AS3 and miR-490-5p. (a) The Venn graph achieved the intersection of MAGI2-AS3 and miR-490-5p, with the differentially expressed genes in TCGA-PAAD analyzed by the GEPIA website (http://gepia.cancer-pku.cn/) and the miR-490-5p targeted lncRNA predicted by the LncBase database (http://carolina.imis.athena-innovation.gr/diana_tools/web/index.php?r=lncbasev2%2Findex). (b) Prediction of target relation between MAGI2-AS3 and miR-490-5p using GEPIA website. (c) The targeting relation between MAGI2-AS3 and miR-490-5p was identified by dual-luciferase reporter gene analysis. (d) The differential expression of MAGI2-AS3 in PAAD was analyzed by GEPIA. (e) Relative expression of MAGI2-AS3 in pancreatic cancer tissues was found to be lower than that in adjacent tissues, which was detected by qRT-PCR. (f) *Pearson* assay manifested that MAGI2-AS3 was negatively associated with miR-490-5p (figure 3f, *p* = 0.020, *r* = −0.516). **p < 0.001, vs. control+MAGI2-AS3-3ʹ-UTR or control; ^^^^*p* < 0.001, vs. miR-490-5p+MAGI2-AS3-3ʹ-UTR-mut.

We applied the GEPIA website (http://gepia.cancer-pku.cn/) to analyze the differentially expressed genes of TCGA-PAAD (pancreatic adenocarcinoma), and the LncBase database (http://carolina.imis.athena-innovation.gr/diana_tools/web/index.php?r=lncbasev2%2Findex) to predict the potential of miR-490-5p to target lncRNA. The two were crossed by the Venn graph, and MAGI2-AS3, ranked number one, was selected as the research object (Figure 3a). Figure 3b exhibited the binding sites of miR-490-5p and MAGI2-AS3 predicted by the GEPIA website. The dual-luciferase verification experiment proved that the fluorescence activity of the miR-490-5p+MAGI2-AS3-3ʹ-UTR group was significantly lower than that of the control group (Figure 3c, *p* < 0.01), thus confirming the combination of the two. According to the GEPIA analysis, MAGI2-AS3 was highly expressed in PAAD patients (Figure 3d, *p* < 0.05), which was consistent with our verification tests. In the collected clinical samples, the expression of MAGI2-AS3 in pancreatic cancer tissues was significantly higher than that in adjacent tissues (Figure 3e, *p* < 0.01). *Pearson* assay manifested that MAGI2-AS3 was negatively associated with miR-490-5p (figure 3f, *p* = 0.020, *r* = −0.516).

### The effect of miR-490-5p on PANC-1 cells could be partly reversed by MAGI2-AS3

**Figure 4.**
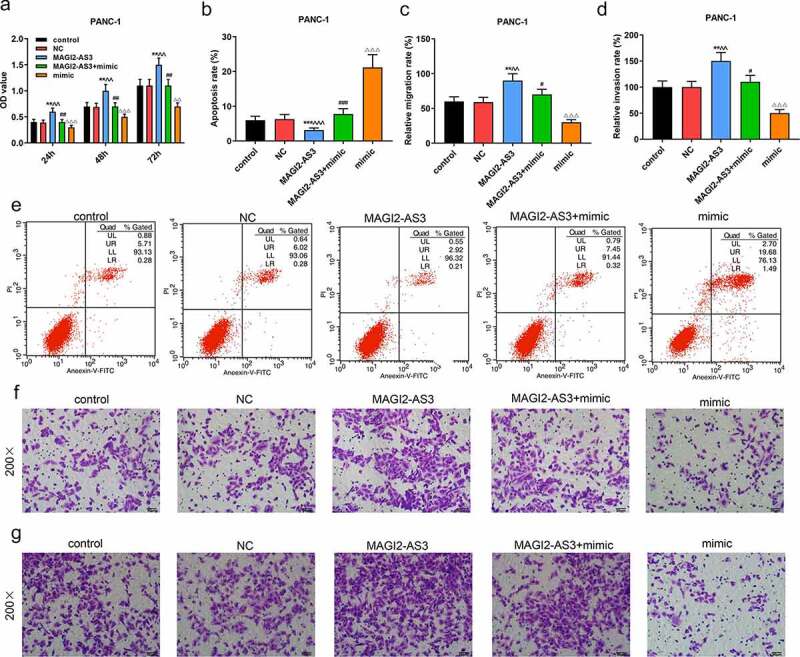
MiR-490-5p targeting MAGI2-AS3 to regulate cell proliferation, migration, apoptosis and invasion in rescue experiments. (a) MAGI2-AS3 partially reversed the inhibitory effect of miR-490-5p overexpression on cell proliferation, which was detected by CCK-8. (b, e) MAGI2-AS3 partially reversed the enhanced effect of miR-490-5p overexpression on cell apoptosis, which was detected by flow cytometry. (c, f) Overexpression of miR-490-5p inhibited cell migration and MAGI2-AS3 partially reversed this effect, which was detected by transwell experiment. (d, g) MAGI2-AS3 partially reversed the inhibitory effect of miR-490-5p overexpression on cell invasion, which was detected by transwell experiment. **p < 0.001, vs. control; ^^^^*p* < 0.001 vs. NC; ^##^P < 0.001, vs. MAGI2-AS3; ^ΔΔ^*p*<0.001, vs MAGI2-AS3+ mimic.

**Figure 5.**
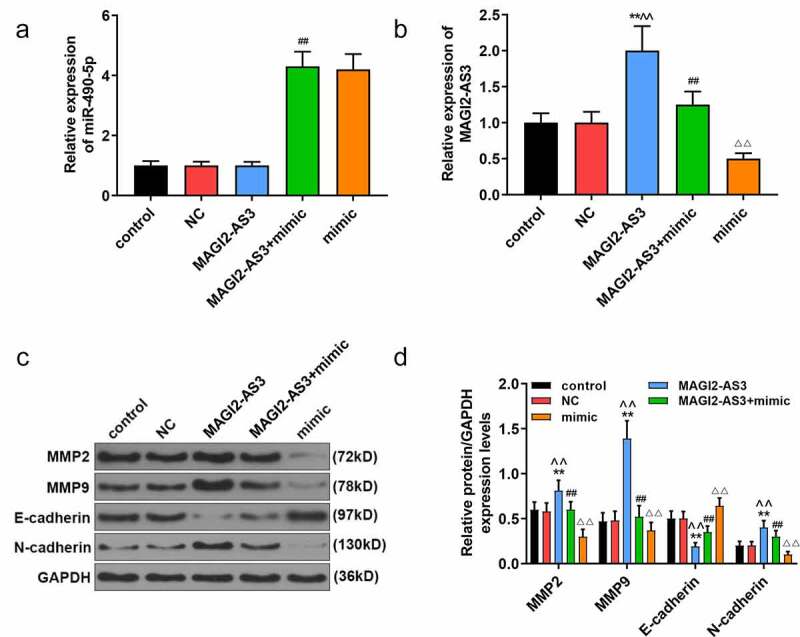
MiR-490-5p targeting MAGI2-AS3 to regulate epithelial–mesenchymal transition (EMT) process. (a) Overexpression of miR-490-5p in cells caused by transfection of miR-490-5p mimic. (b) Up-regulation of miR-490-5p down-regulated the expression of MAGI2-AS3, which was detected by qRT-PCR. (c-d) The mimic group had the lowest protein levels of Matrix metalloproteinase (MMP)2, MMP9 and N-cadherin, and the highest protein level of E-cadherin, while the opposite condition appeared in MAGI2-AS3 group, which were measured by Western blot. **p < 0.001, vs. control; ^^^^*p* < 0.001 vs. NC; ^##^P < 0.001, vs. MAGI2-AS3; ^ΔΔ^*p*<0.001, vs MAGI2-AS3+ mimic.

Since miR-490-5p was most notably under-expressed in PANC-1 cells, and the effect of miR-490-5p mimic on pancreatic cancer was performed in PANC-1 cells, we chose PANC-1 cells to further investigate the role and mechanism of miR-490-5p and MAGI2-AS3 on pancreatic cancer. In line with CCK-8 assay, we noticed that the inhibitory effect of miR-490-5p mimic on proliferation of PANC-1 cells was partly reversed by MAGI2-AS3 (Figure 4a, *p* < 0.01). In apoptosis assay, data demonstrated that overexpression of MAGI2-AS3 induced lower apoptosis rate in MAGI2-AS3 group in PANC-1 cells compared with that in NC group; and it also partly reversed the accelerated effect of miR-490-5p overexpression on PANC-1 cell (Figure 4b and 4e, p<0.01). Additionally, migration and invasion rates of PANC-1 cells were higher in MAGI2-AS3 group, while those in mimic group were decreased compared with those in control and NC groups. And the inhibitory effect of miR-490-5p mimic was partly overturned by MAGI2-AS3 overexpression (Figure 4c-d and 4f-g, *p* < 0.01). Then we measured the mRNA levels of miR-490-5p and MAGI2-AS3, as well as the EMT-related proteins. The results revealed that overexpression of MAGI2-AS3 exerted no effect on miR-490-5p level, while miR-490-5p mimic notably down-regulated MAGI2-AS3 level (Figure 5a-b, *p* < 0.01). In addition, overexpression of MAGI2-AS3 reversed the inhibition of MMP2, MMP9 and N-cadherin, and promotion of E-cadherin in PANC-1 cell in mimic group (Figure 5c-d, *p* < 0.01).

## Discussion

In this study, miR-490-5p was abnormally low expressed in pancreatic cancer, whose inhibitor generated enhanced effects on pancreatic cancer cell biological behaviors as well as the expression of MAGI2-AS3. More importantly, MAGI2-AS3 overexpression partially offset the regulation of miR-490-5p mimic in pancreatic cancer cells, revealing that miR-490-5p played an anti-cancer role through the target gene MAGI2-AS3, which might shed new light on treatment strategy toward pancreatic cancer.

MiRNAs, known as a series of small non-coding RNA molecules (typically 21–25 nucleotides) that regulate gene expression as oncogenes or tumor suppressors in cancers, are considered as useful biomarkers for various human cancers [20]. Previous studies found that miR-210 was significantly low-expressed in gemcitabine-resistant cells, whose overexpression was proved to have a toxic effect on gemcitabine-resistant cells and enhance the sensitivity of gemcitabine, and the mechanism might be the induction of caspase-3-mediated apoptosis and the inhibition of cell cloning process [21]. In another research, expression of the other miRNA (miR-101-3p) was down-regulated in pancreatic cancer, which targeted RRM1 to reverse gemcitabine resistance in the cancer [22]. Moreover, considering that the metastasis is a major cause of cancer-related death, Yoshiro Chijiiwa et al. proposed that miR-5100 overexpression, which was resistant to metastasis, inhibited the pancreatic cancer cell colony formation, cell migration and invasion by directly targeting podxl. And the expression of podxl in pancreatic cancer tissues was related to liver metastasis. These results indicated that miR-5100 and podxl may be potential indicators of pancreatic cancer metastasis with considerable therapeutic potential for anti-metastasis [23]. According to other research, by testing the levels of prognostic biomarkers for pancreatic cancer, patients can be divided into different tumor subgroups treated with personalized anticancer therapy, which may improve survival [24]. Based on studies listed above or more relevant research about pancreatic cancer, functional analysis of miRNAs has aroused substantial research interests.

We, therefore, selected miR-490-5p and conducted experiments to analyze its role in pancreatic cancer. From a recent study, overexpressed miR-490-5p was fatal to pharyngolaryngeal cancer cells by reducing MAP3K9 expression [19]. Another investigation manifested that the expression of miR-490-5p was remarkably down-regulated in the tumor tissues of 30 patients with bladder cancer after surgical resection, compared with that in the adjacent tissues. Further studies in this research documented that the overexpression of miR-490-5p in bladder cancer cells could cause the inhibition of proliferation and the promotion of apoptosis [25]. In this paper, we proved that miR-490-5p was lowly expressed in pancreatic cancer tissues than that in adjacent tissues, which might regulate the biological characteristics of pancreatic cancer cells. To verify this assumption, we further explored the function of miR-490-5p, corroborating that low expression of miR-490-5p promoted the viability, migration and invasion of pancreatic tumor cells and inhibited the apoptosis, while high expression of miR-490-5p produced the opposite effects.

In order to further explore the mechanism of miR-490-5p on pancreatic cancer cells, we applied the GEPIA to analyze the differentially expressed genes of TCGA-PAAD, and the LncBase to predict the potential of miR-490-5p to target lncRNA. The two were crossed by the Venn graph, and MAGI2-AS3, ranked number one, was selected as the research object. MAGI2-AS3 is a newly discovered lncRNA in recent years, which has been confirmed to be involved in the progression of various tumors. Li [26] pointed out that the up-regulation of MAGI2-AS3 is beneficial to the progression of gastric cancer; Liu [17] put forward that the key to the involvement of MAGI2-AS3 in the progression of cervical squamous cell carcinoma lies in the activation of CDK6; and Hao [27] revealed that MAGI2-AS3 restrains malignant phenotype of non-small cell lung cancer by miRNA-23a-3p/PTEN axis. However, there seems to be no accessible literature with respect to MAGI2-AS3 in pancreatic cancer, but it would be conducive to recognizing its role in pancreatic cancer. Then we identified that MAGI2-AS3 was related to the progression of biological behavior in pancreatic cancer. The rescue experiment results in our research indicated that the effect of miR-490-5p overexpression could be partly reversed by MAGI2-AS3and the underlying mechanism may be realized through regulating the progress of EMT.

A study has disclosed that EMT activation was achieved through a variety of related signals, as well as the action of EMT-related transcription factors, which may cause cells to lose epithelialization and gain invasiveness through the progression of EMT. This study also indicated that EMT may have been presented in pancreatic cancer cells [28]. In addition, EMT was reported to be involved in several other cancers, such as hepatocellular carcinoma [29], papillary thyroid carcinoma [30], non-small cell lung cancer [31], colorectal cancer [32], and bladder cancer [33]. MMP2 and MMP9 were EMT-related genes which were closely related to the invasion or metastasis of cancer [34–36]. Moreover, both E-cadherin and N-cadherin were commonly used to detect EMT [37–39]. Different from previous studies, this study demonstrated for the first time that miR-490-5p mimic attenuated the levels of MMP2, MMP9, and N-cadherin and enhanced E-cadherin level in pancreatic cancer cells, which was reversed by MAGI2-AS3 overexpression.

However, what is less clear in our study refers to the role of MAGI2-AS3 in animal experiments, and whether miR-490-5p targeting MAGI2-AS3 regulates EMT through the relevant signaling pathway.

## Conclusion

Taken together, our data proved that miR-490-5p mimic weakened the malignant phenotype of pancreatic cancer through regulating EMT via attenuating MAGI2-AS3, which offered a new entry point to the therapy of pancreatic cancer.

## Highlight

- 1. MiR-490-5p had abnormally low expression in pancreatic cancer tissues and cells.
- 2. MiR-490-5p inhibitor augmented the malignant phenotype of pancreatic cancer.
- 3. MAGI2-AS3 was highly expressed in pancreatic cancer tissues.
- 4. MiR-490-5p acted as an anti-cancer role in pancreatic cancer by targeting MAGI2-AS3.
